# Polar Grid Navigation Algorithm for Unmanned Underwater Vehicles

**DOI:** 10.3390/s17071599

**Published:** 2017-07-09

**Authors:** Zheping Yan, Lu Wang, Wei Zhang, Jiajia Zhou, Man Wang

**Affiliations:** Marine Assembly and Automatic Technology Institute, College of Automation, Harbin Engineering University; Harbin 150001, China; yanzheping@hrbeu.edu.cn (Z.Y.); dawizw@163.com (W.Z.); zhoujiajia@hrbeu.edu.cn (J.Z.); wangmanheu@foxmail.com (M.W.)

**Keywords:** unmanned underwater vehicle (UUV), grid frame, modified adaptive Kalman filter, T-S fuzzy logic, the polar region

## Abstract

To solve the unavailability of a traditional strapdown inertial navigation system (SINS) for unmanned underwater vehicles (UUVs) in the polar region, a polar grid navigation algorithm for UUVs is proposed in this paper. Precise navigation is the basis for UUVs to complete missions. The rapid convergence of Earth meridians and the serious polar environment make it difficult to establish the true heading of the UUV at a particular instant. Traditional SINS and traditional representation of position are not suitable in the polar region. Due to the restrictions of the complex underwater conditions in the polar region, a SINS based on the grid frame with the assistance of the OCTANS and the Doppler velocity log (DVL) is chosen for a UUV navigating in the polar region. Data fusion of the integrated navigation system is realized by a modified fuzzy adaptive Kalman filter (MFAKF). By neglecting the negative terms, and using T-S fuzzy logic in the adaptive regulation of the noise covariance, the proposed filter algorithm can improve navigation accuracy. Simulation and experimental results demonstrate that the polar grid navigation algorithm can effectively navigate a UUV sailing in the polar region.

## 1. Introduction

Unmanned underwater vehicles (UUVs) have been widely used in underwater resource exploration, military reconnaissance, marine surveying, and marine mapping [1,2]. Without the operation of humans, UUVs can carry out the missions autonomously. With the rapid development of underwater navigation technology, humans can explore polar underwater resources through UUVs.

Precise navigation is the premise and basis for UUV sailing. A large number of navigation algorithms have been proposed for UUVs. According to the principle, UUV navigation can be divided into acoustic navigation, visual navigation, inertial navigation, radio navigation, satellite navigation, and so on. Inertial navigation is the most widely used because of its high autonomy. Filtering methods are another important factor that affects navigation accuracy. Filters are widely used in data fusion, such as the Kalman filter (KF), extended Kalman filter (EKF) [3,4], unscented Kalman filter (UKF) [5], and adaptive Kalman filter (AKF). A number of successful tests have also been conducted on these navigation strategies, such as the off-line verification of the UKF-based navigation algorithm for the Typhoon AUV [6]. Cooperative navigation is an effective strategy for efficiently performing tasks while reducing costs. It is also a significant approach for solving the navigation problem in middle-depth of underwater conditions [7]. Algorithms proposed to realize the cooperative navigation include tetrahedral configuration [8], measurement distribution framework [9], and so on. Scholars have done a great deal of research for UUV navigating in the non-polar region and have made a large number of encouraging achievements.

Different from the navigation in the non-polar areas [10], some common navigation methods cannot be applied in the polar region. There are restrictions from the serious environment and the complex underwater conditions in the polar region. As radio signals cannot be spread underwater, Global Positioning System (GPS) is unavailable for UUVs. Meanwhile, the rapid convergence of Earth meridians makes it difficult to establish the true heading of the UUV in the polar region [11]. Therefore, a strapdown inertial navigation system (SINS), which is highly autonomous and stable, becomes the first choice for UUV navigating in the polar region [12]. However, there are some difficulties in the application of the traditional north-oriented SINS algorithm in the polar region, such as calculation overflow and errors increasing sharply. Due to the rapid convergence of Earth meridians, small measurement errors would lead to significant calculation errors. To solve these problems, a transversal SINS algorithm is proposed to replace the traditional SINS algorithm in the polar region [13]. Although a system reset is used to restrain the error drifts, there are principle errors in the transversal SINS algorithm. The transversal SINS ignores the ellipse of the Earth.

Therefore, it is important to choose an available form of SINS for UUVs navigating in the polar region. The polar grid navigation algorithm, which has already helped large aircraft flying though the polar region [14], is the best choice. Selecting the Greenwich meridian as the reference line, the polar grid navigation algorithm can solve the problems caused by the meridians’ convergence [15]. The definition of the grid frame has been given in [16,17] briefly. However, the detailed definition and the derivation of the SINS are not proposed in these papers. The error of approximating a large circle to a straight line in the grid frame is discussed in [18]. The attitude differential equation based on the grid frame is derived in [19]. Although some aspects of the navigation algorithm based on the grid frame are analyzed in the above papers, the complete error model and the filter model are not established. Additionally, these papers did not combine with the specific objects and some specific characters were not taken into consideration. Only in [14,20] was the navigation algorithm based on the grid frame combined with a large aircraft. Different from the flight environment of large aircraft, which has high speed, high precision, and auxiliary forms of a variety of external information, the underwater environment of the UUV is more complex with a lack of radio signal, low visibility, and high requirements of concealment. This navigation algorithm cannot be used on UUVs directly. Humans can help to make decisions during aircraft flights, while UUVs sail alone without the help of humans. The underwater environment is more complex than that of air. For example, large aircraft are affected by wind while UUVs are affected by ocean waves, ocean currents, etc. Therefore, an integrated navigation algorithm is needed to improve the navigation accuracy. Based on the analysis above, the polar grid navigation algorithm for UUVs is proposed considering the underwater environment and the characters of UUV.

In order to improve the navigation accuracy, the OCTANS and the Doppler velocity log (DVL) are chosen as the assistants for the long-endurance navigation of UUVs in the polar region. Combined with the SINS, a SINS/OCTANS/DVL integrated navigation system is constituted. Meanwhile, data fusion of the integrated navigation system is realized by a modified fuzzy adaptive Kalman filter (MFAKF).

In this paper, a polar grid navigation algorithm for UUVs is proposed. Based on the grid frame, the SINS is used for navigating the UUV in the polar region. With the assistance of the OCTANS and DVL, a SINS/OCTANS/DVL integrated navigation system is established. The main contribution of this paper is the derivation of the complete error model and filter model of the polar grid navigation based on the characters of the UUV and the proposed modified AKF to improve the navigation accuracy of the UUV. The following sections are arranged as follows: Error equations and filter models are deduced in Section 2 and Section 3, respectively; In Section 4, a modified fuzzy adaptive Kalman filter is proposed; The results of simulations and experiments are expressed in Section 5; In Section 6, the results and error sources are discussed; Finally, the conclusions are drawn in Section 7.

## 2. Error Equations of the UUV in Polar Grid Navigation

Frames are important for navigation. The transformation relations among frames can reflect motions of the UUV. In this paper, *i* frame, *e* frame, *g* frame, *G* frame, *b* frame, *n* frame, *o* frame, and *m* frame represent the inertial frame, Earth-centered Earth-fixed frame, geographic frame, grid frame, body frame of the UUV, navigation frame, body frame of the OCTANS, and the body frame of DVL, respectively. The meridians’ convergence in high-latitude areas has no influence on the grid frame. Therefore, the grid frame (*G* frame) is chosen as the navigation frame (*n* frame).

Error equations of the UUV consist of the attitude error equation, velocity error equation, and position error equation. The main error sources of the SINS are from inertial measurement unit (IMU), which is composed of gyroscopes and accelerometers. Their influence on navigation can be obtained from the following error equations. In addition, error models of the OCTANS and DVL are also established in this section.

### 2.1. Grid Frame (G Frame)

**Figure 1:**
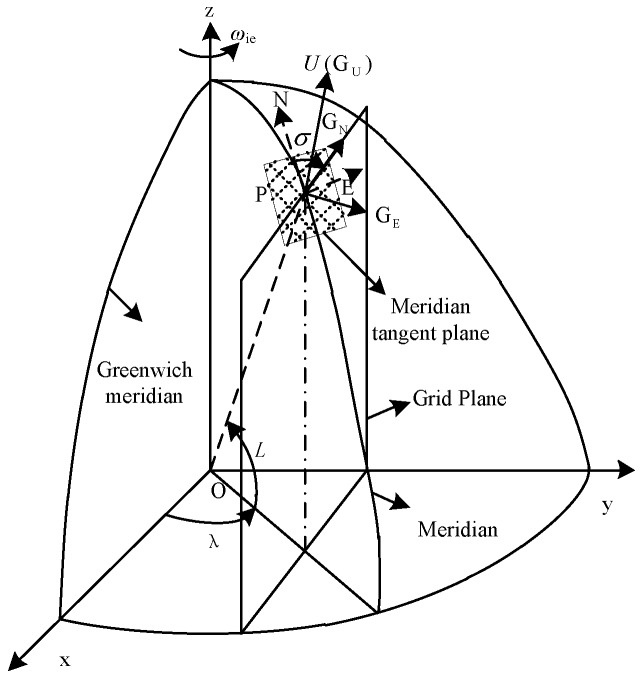
Definition of the grid frame.

In Figure 1, point P represents the position of the UUV. The grid planes are the planes that are parallel with the Greenwich plane. The grid plane of point P is the grid plane passes through point P. The grid north axis lies along the intersection of the grid plane and the tangent plane of the earth passing point P. The grid up axis and the geographic up axis coincide. The grid east axis, grid north axis, and grid up axis constitute the right-handed frame that is the grid frame. There is an angle σ between the grid north axis and the geographic north axis.

As the grid up axis coincides with the geographic up axis, the grid frame can be obtained from the geographic frame by rotating σ around the up axis [15]. Therefore, the transform relations among the g frame, G frame, and e frame can be described as:

(1) $$C_{g}^{G}=\left[\begin{array}{ccc} \cos\sigma & -\sin\sigma & 0\\ \sin\sigma & \cos\sigma & 0\\ 0 & 0 & 1 \end{array}\right],$$

(2) $$C_{e}^{g}=\left[\begin{array}{ccc} -\sin\lambda & \cos\lambda & 0\\ -\sin L\cos\lambda & -\sin L\sin\lambda & \cos L\\ \cos L\cos\lambda & \cos L\sin\lambda & \sin L \end{array}\right],$$

(3) $$C_{e}^{G}=C_{e}^{g}C_{g}^{G}=\left[\begin{array}{ccc} -c\sigma s\lambda +s\sigma c\lambda sL & c\sigma c\lambda +s\sigma sLs\lambda & -s\sigma cL\\ -s\sigma s\lambda -c\sigma sLc\lambda & s\sigma c\lambda -c\sigma sLs\lambda & c\sigma cL\\ cLc\lambda & cLs\lambda & sL \end{array}\right],$$

The angle σ can be expressed as:

(4) $$\sin\sigma =\frac{\sin\lambda \sin L}{\sqrt{1-\cos^{2}L\sin^{2}\lambda }},$$

(5) $$\cos\sigma =\frac{\cos\lambda }{\sqrt{1-\cos^{2}L\sin^{2}\lambda }},$$

The one-order increment of Equation (5) can be written as:

(6) $$\delta \sigma =\frac{\sin L}{1-\cos^{2}L\sin^{2}\lambda }\delta \lambda +\frac{\sin\lambda cos\lambda cosL}{1-\cos^{2}L\sin^{2}\lambda }\delta L,$$

By substituting Equations (4)–(5) into Equation (3), $C_{e}^{G}$ can be rewritten as:

(7) $$C_{e}^{G}=\left[\begin{array}{ccc} \frac{-c^{2}Ls\lambda \;c\lambda }{\sqrt{1-c^{2}Ls^{2}\lambda }} & \sqrt{1-c^{2}Ls^{2}\lambda } & \frac{-s\lambda sLcL}{\sqrt{1-c^{2}Ls^{2}\lambda }}\\ \frac{-sL}{\sqrt{1-c^{2}Ls^{2}\lambda }} & 0 & \frac{c\lambda cL}{\sqrt{1-c^{2}Ls^{2}\lambda }}\\ cLc\lambda & cLs\lambda & sL \end{array}\right],$$

Due to the rapid convergence of Earth meridians in high-latitude areas, small position errors will lead a large divergence in longitude. Thus, latitude and longitude are not suitable to describe the position of the UUV in high-latitude areas. In this paper, the coordinate in e frame $R^{e}\left(x,y,z\right)$ is chosen to express the position of the UUV. Latitude and longitude of the position can be obtained from $R^{e}\left(x,y,z\right)$:

(8) $$\left\{ \begin{array}{l} x=R_{Nh}\cos L\cos\lambda \\ y=R_{Nh}\cos L\sin\lambda \\ z=[R_{N}(1-e^{2})+h]\sin L \end{array}\right.,$$

(9) $$x^{2}+y^{2}={\left(R_{Nh}\cos L\right)}^{2},$$

Then,

(10) $$\cos L=\frac{\sqrt{x^{2}+y^{2}}}{R_{Nh}},$$

(11) $$\sin L=\pm \sqrt{1-\frac{x^{2}+y^{2}}{R_{Nh}^{2}}},$$

(12) $$\sin\lambda =\frac{y}{R_{Nh}\cos L}=\frac{y}{\sqrt{x^{2}+y^{2}}},$$

(13) $$\cos\lambda =\frac{x}{R_{Nh}\cos L}=\frac{x}{\sqrt{x^{2}+y^{2}}},$$

The longitude λ can be calculated from Equations (12) and (13) directly, while the calculation of latitude L needs the approximate solution [21].

Ignoring the ellipse of the earth, the radius of the earth is approximate to $R_{Mh}\approx R_{Nh}\approx R_{eh}$.

(14) $$\left\{ \begin{array}{l} x=R_{eh}\cos L\cos\lambda \\ y=R_{eh}\cos L\sin\lambda \\ z=R_{eh}\sin L \end{array}\right.,$$

The one-order increment of Equation (14) can be described as:

(15) $$\left\{ \begin{array}{l} \delta x=-R_{eh}sLc\lambda \delta L-R_{eh}cLs\lambda \delta \lambda +cLc\lambda \delta h\\ \delta y=-R_{eh}sLs\lambda \delta L+R_{eh}cLc\lambda \delta \lambda +cLs\lambda \delta h\\ \delta z=R_{eh}cL\delta L+sL\delta h \end{array}\right.,$$

(16) $$\delta R^{e}=\left[\begin{array}{c} \delta x\\ \delta y\\ \delta z \end{array}\right]=\left[\begin{array}{ccc} -R_{eh}\sin L\cos\lambda & -R_{eh}\cos L\sin\lambda & \cos L\cos\lambda \\ -R_{eh}\sin L\sin\lambda & R_{eh}\cos Lc\lambda & \cos L\sin\lambda \\ R_{eh}\cos L & 0 & \sin L \end{array}\right]\left[\begin{array}{c} \delta L\\ \delta \lambda \\ \delta h \end{array}\right],$$

Thus,

(17) $$\delta P=\left[\begin{array}{c} \delta L\\ \delta \lambda \\ \delta h \end{array}\right]=\frac{1}{R_{eh}}\left[\begin{array}{ccc} -\sin Lc\lambda & -\sin L\sin\lambda & \cos L\\ -\frac{\sin\lambda }{\cos L} & \frac{\cos\lambda }{\cos L} & 0\\ R_{eh}\cos L\cos\lambda & R_{eh}\cos L\sin\lambda & R_{eh}\sin L \end{array}\right]\delta R^{e},$$

### 2.2. Attitude Error Equation

Due to the restriction of the computer and algorithm, there are errors between the ideal *G* frame and the actual *G* frame. The actual *G* frame in practical application of the SINS is defined as the *G*’ frame. The attitude differential equation of the UUV in ideal conditions can be expressed as:

(18) $${\dot{C}}_{b}^{G}=C_{b}^{G}\left[\omega_{ib}^{b}\times \right]-\left[\omega_{iG}^{G}\times \right]C_{b}^{G},$$

where $\left[\omega_{iG}^{G}\times \right]$ is the anti-symmetric matrix of $\omega_{iG}^{G}$, which can be expressed as:

(19) $$\left[\omega_{iG}^{G}\times \right]=\left[\omega_{ie}^{G}\times \right]+\left[\omega_{eG}^{G}\times \right],$$

(20) $$\omega_{ie}^{G}=C_{g}^{G}\omega_{ie}^{g}=\left[\begin{array}{ccc} \cos\sigma & -\sin\sigma & 0\\ \sin\sigma & \cos\sigma & 0\\ 0 & 0 & 1 \end{array}\right]\left[\begin{array}{c} 0\\ \omega_{ie}\cos L\\ \omega_{ie}\sin L \end{array}\right]=\left[\begin{array}{c} -\omega_{ie}\cos L\sin\sigma \\ \omega_{ie}\cos L\cos\sigma \\ \omega_{ie}\sin L \end{array}\right],$$

(21) $$\omega_{eG}^{G}=\left[\begin{array}{c} \omega_{eGx}^{G}\\ \omega_{eGy}^{G}\\ \omega_{eGz}^{G} \end{array}\right]=\left[\begin{array}{cc} \frac{1}{\tau_{fG}} & -\frac{1}{R_{yG}}\\ \frac{1}{R_{xG}} & -\frac{1}{\tau_{fG}}\\ \frac{K_{G}}{\tau_{fG}} & -\frac{K_{G}}{R_{yG}} \end{array}\right]\left[\begin{array}{c} v_{G_{E}}\\ v_{G_{N}} \end{array}\right],$$

where $\frac{1}{R_{xG}}=\frac{\sin^{2}\sigma }{R_{Mh}}+\frac{\cos^{2}\sigma }{R_{Nh}}$ and $\frac{1}{R_{yG}}=\frac{\cos^{2}\sigma }{R_{Mh}}+\frac{\sin^{2}\sigma }{R_{Nh}}$ are the equivalent curvature; $\frac{1}{\tau_{fG}}=\left(\frac{1}{R_{Mh}}-\frac{1}{R_{Nh}}\right)\sin\sigma \cos\sigma$ is the twist rate of the ellipsoid at point *P*, and $K_{G}=\frac{\sin\lambda \cos L}{\sqrt{1-\cos^{2}L\sin^{2}\lambda }}=\frac{\sin\lambda \sin L\cos L}{\sin L\sqrt{1-\cos^{2}L\sin^{2}\lambda }}=\frac{\cos L}{\sin L}\sin\sigma =\cot L\sin\sigma$. Ignoring the ellipse of the Earth and the influence of distortion, $\omega_{eG}^{G}$ can be simplified as:

(22) $$\omega_{eG}^{G}=\left[\begin{array}{cc} 0 & -\frac{1}{R_{eh}}\\ \frac{1}{R_{eh}} & 0\\ 0 & -\frac{K_{G}}{R_{eh}} \end{array}\right]\left[\begin{array}{c} v_{G_{E}}\\ v_{G_{N}} \end{array}\right]=\left[\begin{array}{c} -\frac{v_{GN}}{R_{eh}}\\ \frac{v_{GE}}{R_{eh}}\\ -\frac{v_{GN}}{R_{eh}}\cot Ls\sigma \end{array}\right],$$

Due to the errors in practical application, the attitude differential equation of UUV can be written as:

(23) $${\dot{C}}_{b}^{G^{\prime }}=C_{b}^{G^{\prime }}\left[{\hat{\omega }}_{ib}^{b}\times \right]-\left[{\hat{\omega }}_{iG}^{G}\times \right]C_{b}^{G^{\prime }},$$

where:

(24) $${\hat{\omega }}_{ib}^{b}=\omega_{ib}^{b}+\delta \omega_{ib}^{b},$$

(25) $${\hat{\omega }}_{iG}^{G}={\hat{\omega }}_{ie}^{G}+{\hat{\omega }}_{eG}^{G},$$

(26) $${\hat{\omega }}_{ie}^{G}=\omega_{ie}^{G}+\delta \omega_{ie}^{G},$$

(27) $${\hat{\omega }}_{eG}^{G}=\omega_{eG}^{G}+\delta \omega_{eG}^{G},$$

where $\delta \omega_{ib}^{b}$, $\delta \omega_{ie}^{G}$, and $\delta \omega_{eG}^{G}$ are the errors of $\omega_{ib}^{b}$, $\omega_{ie}^{G}$, and $\omega_{eG}^{G}$, respectively. $\delta \omega_{ie}^{G}$ and $\delta \omega_{eG}^{G}$ can be defined as:

(28) $$\delta \omega_{ie}^{G}={\hat{\omega }}_{ie}^{G}-\omega_{ie}^{G}=\omega_{ie}\left[\begin{array}{c} -\cos L\cos\sigma \\ -\cos L\sin\sigma \\ 0 \end{array}\right]\delta \sigma +\omega_{ie}\left[\begin{array}{c} \sin L\sin\sigma \\ -\sin L\cos\sigma \\ \cos L \end{array}\right]\delta L,$$

(29) $$\delta \omega_{eG}^{G}={\hat{\omega }}_{eG}^{G}-\omega_{eG}^{G}=\left[\begin{array}{ccc} 0 & 0 & \frac{v_{GN}}{R_{eh}^{2}}\\ 0 & 0 & -\frac{v_{GE}}{R_{eh}^{2}}\\ \frac{v_{GN}\sin\sigma }{R_{eh}\sin^{2}L} & 0 & \frac{v_{GN}\cot L\sin\sigma }{R_{eh}^{2}} \end{array}\right]\left[\begin{array}{c} \delta L\\ \delta \lambda \\ \delta h \end{array}\right]+\left[\begin{array}{ccc} 0 & -\frac{1}{R_{eh}} & 0\\ \frac{1}{R_{eh}} & 0 & 0\\ 0 & -\frac{\cot Ls\sigma }{R_{eh}} & 0 \end{array}\right]\left[\begin{array}{c} \delta v_{GE}\\ \delta v_{GN}\\ \delta v_{GU} \end{array}\right]+\left[\begin{array}{c} 0\\ 0\\ -\frac{v_{GN}\cot L\cos\sigma }{R_{eh}} \end{array}\right]\delta \sigma ,$$

The velocity error can be described as $\delta V^{G}={\left[\delta v_{GE}\;\delta v_{GN}\;\delta v_{GU}\right]}^{T}$. Then, based on Equation (6), $\delta \omega_{ie}^{G}$ and $\delta \omega_{eG}^{G}$ can be rewritten as:

(30) $$\delta \omega_{ie}^{G}=C_{\omega iep}\delta P,$$

(31) $$\delta \omega_{eG}^{G}=C_{\omega eGp}\delta P+C_{\omega eGv}\delta V^{G},$$

where:

(32) $$C_{\omega iep}=\omega_{ie}\left[\begin{array}{ccc} \sin L\sin\sigma -\cot L\cos L\cos^{2}\sigma \sin\sigma & -\frac{\cot L\cos\sigma \sin^{2}\sigma }{s^{2}\lambda } & 0\\ -\sin L\cos\sigma \;-\cot LcL\sin^{2}\sigma \cos\sigma & -\frac{\cot L\sin^{3}\sigma }{\sin^{2}\lambda } & 0\\ cL & 0 & 0 \end{array}\right],$$

(33) $$C_{\omega eGp}=\left[\begin{array}{ccc} 0 & 0 & \frac{v_{GN}}{R_{eh}^{2}}\\ 0 & 0 & -\frac{v_{GE}}{R_{eh}^{2}}\\ \frac{v_{GN}s\sigma }{R_{eh}(1-c^{2}Lc^{2}\sigma )} & -\frac{v_{GN}cLc\sigma }{R_{eh}(1-c^{2}Ls^{2}\lambda )} & \frac{v_{GN}\cot Ls\sigma }{R_{eh}^{2}} \end{array}\right],$$

(34) $$C_{\omega eGv}=\left[\begin{array}{ccc} 0 & -\frac{1}{R_{eh}} & 0\\ \frac{1}{R_{eh}} & 0 & 0\\ 0 & -\frac{\cot L\sin\sigma }{R_{eh}} & 0 \end{array}\right],$$

By substituting Equation (17) into Equations (30)–(31) and neglecting the small high-order terms, $\delta \omega_{ie}^{G}$ and $\delta \omega_{eG}^{G}$ can be rewritten as:

(35) $$\delta \omega_{ie}^{G}=C_{\omega ieR}\delta R^{e},$$

(36) $$\delta \omega_{eG}^{G}=C_{\omega eGR}\delta R^{e}+C_{\omega eGv}\delta V^{G},$$

where:

(37) $$C_{\omega ieR}=\frac{\omega_{ie}}{R_{eh}{\left(1-c^{2}Ls^{2}\lambda \right)}^{\frac{3}{2}}}\cdot \left[\begin{array}{ccc} 2c^{2}LsLs\lambda c\lambda & -sL\left[c^{2}\lambda +s^{2}\lambda \cdot \left(s^{2}L-c^{2}L\right)\right] & s^{2}LcLs\lambda \\ s^{2}L & 0 & -sLcLc\lambda \\ -sLcLc\lambda & -sLcLs\lambda & c^{2}L \end{array}\right],$$

(38) $$C_{\omega eGR}=\frac{1}{R_{eh}^{2}}\cdot \left[\begin{array}{ccc} v_{GN}cLc\lambda & v_{GN}cLs\lambda & v_{GN}sL\\ -v_{GE}cLc\lambda & -v_{GE}cLs\lambda & -v_{GE}sL\\ \frac{2v_{GN}\;c^{2}Ls\lambda c\lambda }{{\left(1-c^{2}Ls^{2}\lambda \right)}^{\frac{3}{2}}} & -v_{GN}\sqrt{1-c^{2}Ls^{2}\lambda } & \frac{2v_{GN}\;sLcLs\lambda }{{\left(1-c^{2}Ls^{2}\lambda \right)}^{\frac{3}{2}}} \end{array}\right],$$

where s(⋅) and c(⋅) represent sin(⋅) and cos(⋅), respectively.

The attitude error matrix can be defined as:

(39) $$\Delta C=C_{b}^{G^{\prime }}-C_{b}^{G},$$

(40) $$\Delta C=\left(I-C_{G^{\prime }}^{G}\right)C_{b}^{G^{\prime }},$$

By substituting Equation (23) into Equation (40), the derivative of ΔC can be expressed as:

(41) $$\Delta \dot{C}=C_{b}^{G^{\prime }}\left[{\hat{\omega }}_{ib}^{b}\times \right]-\left[{\hat{\omega }}_{iG}^{G}\times \right]C_{b}^{G^{\prime }}-C_{G^{\prime }}^{G}C_{b}^{G^{\prime }}\left[{\hat{\omega }}_{ib}^{b}\times \right]+C_{G^{\prime }}^{G}\left[{\hat{\omega }}_{iG}^{G}\times \right]C_{b}^{G^{\prime }}-{\dot{C}}_{G^{\prime }}^{G}C_{b}^{G^{\prime }},$$

By substituting Equations (18) and (23), $\Delta \dot{C}$ can be rewritten as:

(42) $$\Delta \dot{C}=C_{b}^{G^{\prime }}\left[{\hat{\omega }}_{ib}^{b}\times \right]-\left[{\hat{\omega }}_{iG}^{G}\times \right]C_{b}^{G^{\prime }}-C_{G^{\prime }}^{G}C_{b}^{G^{\prime }}\left[\omega_{ib}^{b}\times \right]+\left[\omega_{iG}^{G}\times \right]C_{G^{\prime }}^{G}C_{b}^{G^{\prime }},$$

Combined Equation (41) and (42):

(43) $$C_{G^{\prime }}^{G}C_{b}^{G^{\prime }}\left[\delta \omega_{ib}^{b}\times \right]+\left[\omega_{iG}^{G}\times \right]C_{G^{\prime }}^{G}C_{b}^{G^{\prime }}-C_{G^{\prime }}^{G}\left[{\hat{\omega }}_{iG}^{G}\times \right]C_{b}^{G^{\prime }}+{\dot{C}}_{G^{\prime }}^{G}C_{b}^{G^{\prime }}=0,$$

Based on the similar transformation theory of matrix, Equation (43) can be simplified as:

(44) $$\delta \omega_{ib}^{G^{\prime }}+\omega_{iG}^{G^{\prime }}-{\hat{\omega }}_{iG}^{G}+\omega_{GG^{\prime }}^{G^{\prime }}=0,$$

Thus,

(45) $$\omega_{GG^{\prime }}^{G^{\prime }}=\left(I-C_{G}^{G^{\prime }}\right)\omega_{iG}^{G}+\delta \omega_{iG}^{G}-C_{b}^{G^{\prime }}\delta \omega_{ib}^{b},$$

The misalignment angle of the UUV between the G frame and $G^{\prime }$ frame is $\phi^{G}={\left[\phi_{x}^{G}\;\phi_{y}^{G}\;\phi_{z}^{G}\right]}^{T}$. Thus, $\omega_{GG^{\prime }}^{G^{\prime }}$ can be rewritten as:

(46) $$\omega_{GG^{\prime }}^{G^{\prime }}=\left[\begin{array}{ccc} c\phi_{y}^{G} & 0 & -s\phi_{y}^{G}c\phi_{x}^{G}\\ 0 & 1 & s\phi_{x}^{G}\\ s\phi_{y}^{G} & 0 & c\phi_{y}^{G}c\phi_{x}^{G} \end{array}\right]\left[\begin{array}{c} {\dot{\phi }}_{x}^{G}\\ {\dot{\phi }}_{y}^{G}\\ {\dot{\phi }}_{z}^{G} \end{array}\right]=C_{\omega }{\dot{\phi }}^{G},$$

where:

(47) $$C_{\omega }=\left[\begin{array}{ccc} \cos\phi_{y}^{G} & 0 & -\sin\phi_{y}^{G}\cos\phi_{x}^{G}\\ 0 & 1 & \sin\phi_{x}^{G}\\ \sin\phi_{y}^{G} & 0 & \cos\phi_{y}^{G}\cos\phi_{x}^{G} \end{array}\right],$$

Thus, based on Equation (45), $\phi^{G}$ can be obtained as:

(48) $${\dot{\phi }}^{G}=C_{\omega }{}^{-1}\omega_{GG^{\prime }}^{G^{\prime }}=C_{\omega }{}^{-1}\left(\left(\phi^{G}\times \right)\omega_{iG}^{G}+\delta \omega_{iG}^{G}-C_{b}^{G^{\prime }}\delta \omega_{ib}^{b}\right),$$

Considering:

(49) $$\delta \omega_{ib}^{b}=\varepsilon^{b},$$

(50) $$\delta \omega_{iG}^{G}=\delta \omega_{ie}^{G}+\delta \omega_{eG}^{G}=\left(C_{\omega ieR}+C_{\omega eGR}\right)\delta R^{e}+C_{\omega eGv}\delta V^{G},$$

where $\varepsilon^{b}$ is the gyro drift that consists of the gyro constant drifts $\varepsilon_{c}^{b}$ and the gyro random drifts $\varepsilon_{w}^{b}$, and $\varepsilon_{w}^{b}$ can be set as zero-mean Gaussian white noise.

Therefore, the attitude error equation of UUV in G frame can be rewritten as:

(51) $${\dot{\phi }}^{G}=C_{\omega }{}^{-1}(-(\omega_{iG}^{G}\times )\phi^{G}+(C_{\omega ieR}+C_{\omega eGR})\delta R^{e}+C_{\omega eGv}\delta V^{G}-C_{b}^{G^{\prime }}\varepsilon^{b}),$$

### 2.3. Velocity Error Equation

The velocity differential equation of the UUV in ideal conditions can be described as:

(52) $${\dot{V}}^{G}=C_{b}^{G}f^{b}-\left(2\omega_{ie}^{G}+\omega_{eG}^{G}\right)\times V^{G}+g^{G},$$

where $f^{b}$ is the special force measured by the SINS.

Due to the errors from the IMU, the velocity differential equation of UUV in practical application can be expressed as:

(53) $${\dot{\hat{V}}}^{G}={\hat{C}}_{b}^{G}{\hat{f}}^{b}-\left(2{\hat{\omega }}_{ie}^{G}+{\hat{\omega }}_{eG}^{G}\right)\times {\hat{V}}^{G}+{\hat{g}}^{G},$$

and:

(54) $$\delta V^{G}={\hat{V}}^{G}-V^{G},$$

(55) $${\hat{C}}_{b}^{G}=C_{G}^{G^{\prime }}C_{b}^{G}=\left(I-\phi^{G}\times \right)C_{b}^{G},$$

(56) $$\nabla^{b}={\hat{f}}^{b}-f^{b},$$

(57) $$\delta \omega_{ie}^{G}={\hat{\omega }}_{ie}^{G}-\omega_{ie}^{G},$$

(58) $$\delta \omega_{eG}^{G}={\hat{\omega }}_{eG}^{G}-\omega_{eG}^{G},$$

(59) $$\delta g^{G}={\hat{g}}^{G}-g^{G},$$

where $\delta V^{G}$, $\nabla^{b}$, $\delta \omega_{ie}^{G}$, $\delta \omega_{eG}^{G}$, and $\delta g^{G}$ are the errors of $V^{G}$, $f^{b}$, $\omega_{ie}^{G}$, $\omega_{eG}^{G}$, and $g^{G}$, respectively. $\nabla^{b}$ is the accelerometer bias which consists of the accelerometer constant bias $\nabla_{c}^{b}$ and the accelerometer random bias $\nabla_{w}^{b}$., and $\nabla_{w}^{b}$ can be set as zero-mean Gaussian white noise [22].

Substituting Equations (54)–(59) into Equation (53), then subtracting Equation (52) from Equation (53) and ignoring the second-order small terms, the velocity error equation can be described as:

(60) $$\delta {\dot{V}}^{G}=-\left(\phi^{G}\times \right)C_{b}^{G}f^{b}+C_{b}^{G}\nabla^{b}-\left(2\omega_{ie}^{G}+\omega_{eG}^{G}\right)\times \delta V^{G}-\left(2\delta \omega_{ie}^{G}+\delta \omega_{eG}^{G}\right)\times V^{G},$$

Considering Equations (35) and (36), the velocity error equation of the UUV in the G frame can be rewritten as:

(61) $$\delta {\dot{V}}^{G}=f^{G}\times \phi^{G}+\left[V^{G}\times C_{\omega eGv}-\left(2\omega_{ie}^{G}+\omega_{eG}^{G}\right)\times \right]\cdot \delta V^{G}+\left[V^{G}\times \left(2C_{\omega ieR}+C_{\omega eGR}\right)\right]\delta R^{e}+C_{b}^{G}\nabla^{b},$$

### 2.4. Position Error Equation

Since latitude and longitude are unsuitable for describing the position of the UUV in high-latitude areas, the coordinate in the e frame $R^{e}\left(x,y,z\right)$ is chosen to describe the position. The position differential equation of the UUV in ideal conditions can be written as:

(62) $${\dot{R}}^{e}=C_{G}^{e}V^{G},$$

Similar to the velocity differential equation, the position differential equation of UUV in actual condition can be described as:

(63) $${\dot{\hat{R}}}^{e}={\hat{C}}_{G}^{e}{\hat{V}}^{G},$$

and:

(64) $$\delta R^{e}={\hat{R}}^{e}-R^{e},$$

(65) $$\;\delta V^{G}={\hat{V}}^{G}-V^{G},$$

(66) $${\hat{C}}_{G}^{e}=C_{G^{\prime \prime }}^{e}=C_{G}^{e}C_{G^{\prime \prime }}^{G},$$

Due to the position deviation, the actual position of the UUV and the calculated position in the G frame do not coincide. There are slight errors δL, δλ, and δσ between them. The G frame can be obtained by the following three-time rotations from the $G^{\prime \prime }$ frame:

(67) $$Ox_{G^{\prime \prime }}y_{G^{\prime \prime }}z_{G^{\prime \prime }}\overset{around\;x_{g}\;axis}{\underset{\delta L}{\to }}Ox_{G^{\prime \prime }}^{\prime }y_{G^{\prime \prime }}^{\prime }z_{G^{\prime \prime }}^{\prime }\overset{around\;z_{e}\;axis}{\underset{-\delta \lambda }{\to }}Ox_{G^{\prime \prime }}^{\prime \prime }y_{G^{\prime \prime }}^{\prime \prime }z_{G^{\prime \prime }}^{\prime \prime }\overset{around\;{\hat{z}}_{G}\;axis}{\underset{\delta \sigma }{\to }}Ox_{G}y_{G}z_{G},$$

The position deviation can be expressed as:

(68) $$\delta \beta =\delta Lx_{g}-\delta \lambda z_{e}+\delta \sigma {\hat{z}}_{G},$$

Ignoring the second-order small terms and combining Equation (6), Equation (68) is transformed into the matrix form based on G frame.

(69) $$\delta \beta^{G}=\left[\begin{array}{ccc} -\cos\sigma & -\cos L\sin\sigma & 0\\ -\sin\sigma & \cos L\cos\sigma & 0\\ -\frac{\cos L\cos\sigma \sin\sigma }{\sin L} & \frac{\cos\sigma \sin\sigma }{\sin\lambda \cos\lambda }+\sin L & 0 \end{array}\right]\delta P,$$

(70) $$\delta \beta^{G}=C_{\beta R}\delta R^{e},$$

where,

(71) $$C_{\beta R}=\frac{1}{R_{eh}}\left[\begin{array}{ccc} \frac{sL}{\sqrt{1-c^{2}Ls^{2}\lambda }} & 0 & \frac{-c\lambda cL}{\sqrt{1-c^{2}Ls^{2}\lambda }}\\ \frac{s\lambda c\lambda c^{2}L}{\sqrt{1-c^{2}Ls^{2}\lambda }} & \sqrt{1-c^{2}Ls^{2}\lambda } & \frac{-s\lambda sLcL}{\sqrt{1-c^{2}Ls^{2}\lambda }}\\ \frac{cLsLs\lambda }{1-c^{2}Ls^{2}\lambda } & 0 & -\;\frac{c^{2}Ls\lambda c\lambda }{1-c^{2}Ls^{2}\lambda } \end{array}\right],$$

Thus:

(72) $${\hat{C}}_{G}^{e}=C_{G}^{e}C_{G^{\prime }}^{G}=C_{G}^{e}\left(I+\delta \beta^{G}\times \right),$$

Substituting Equations (64)–(66) and Equation (72) into Equation (63), then subtracting Equation (62) from Equation (63), the position error equation of the UUV in the grid frame can be obtained as:

(73) $$\delta {\dot{R}}^{e}=C_{G}^{e}\delta V^{G}-C_{G}^{e}\left(V^{G}\times \right)C_{\beta R}\delta R^{e},$$

### 2.5. Error Model of OCTANS

The OCTANS is an all-in-one gyrocompass and motion sensor for diverse challenging applications. OCTANS consists of three fiber-optic gyroscopes and three quartz accelerometers. The OCTANS measures the attitude of UUV quickly and accurately. The errors of the OCTANS are caused by the inertially-sensitive elements, including gyroscope drifts and accelerometer bias. The accelerometer bias has little effect on the measurement results comparing with the gyroscope drifts. Therefore, the accelerometer bias can be ignored. The gyro drifts consist of gyro constant drifts and gyro random drifts. Therefore, the error model of OCTANS can be built as follows:

(74) $$\varepsilon_{o}^{o}=\varepsilon_{wo}^{o}+\varepsilon_{co}^{o},$$

where $\varepsilon_{co}^{o}$ and $\varepsilon_{wo}^{o}$ are the gyro constant drifts and gyro random drifts of the OCTANS in the o frame, respectively. $\varepsilon_{wo}^{o}$ can be set as zero-mean Gaussian white noise and $\varepsilon_{co}^{o}$ can be expressed as:

(75) $${\dot{\varepsilon }}_{co}^{o}=0,$$

The error model of the OCTANS expressed in the G frame can be rewritten as:

(76) $$\varepsilon_{o}^{G}=C_{b}^{G}C_{o}^{b}\varepsilon_{o}^{o}=C_{b}^{G}C_{o}^{b}\left(\varepsilon_{wo}^{o}+\varepsilon_{co}^{o}\right),$$

where $C_{o}^{b}$ is the direction cosine matrix from the o frame to the b frame. The installation error angles of the OCTANS are small enough to be ignored. Then, $C_{o}^{b}$ is approximate to $C_{o}^{b}=I$. Equation (76) can be rewritten as:

(77) $$\varepsilon_{o}^{G}=C_{b}^{G}I\varepsilon_{o}^{o}=C_{b}^{G}I\left(\varepsilon_{wo}^{o}+\varepsilon_{co}^{o}\right)=\varepsilon_{wo}^{G}+\varepsilon_{co}^{G},$$

Considering $\varepsilon_{co}^{o}$ can be updated by Equation (75), the attitude of the UUV measured by the OCTANS in the G frame can be obtained as:

(78) $${\hat{\Lambda }}_{o}^{G}=\Lambda_{o}^{G}+\varepsilon_{wo}^{G}={\tilde{\Lambda }}_{o}^{G}-\varepsilon_{co}^{G},$$

where $\Lambda ={\left[\varphi \;\theta \;\psi \right]}^{T}$ represents the attitude of the UUV; φ, θ, and ψ represent the roll angle, pitch angle, and yaw angle of the UUV, respectively; ${\hat{\Lambda }}_{o}^{G}$ is the attitude measured by the OCTANS after partial error compensation in G frame; ${\tilde{\Lambda }}_{o}^{G}$ is the attitude measured by the OCTANS before partial error compensation in the G frame; $\Lambda_{o}^{G}$ is the ideal attitude measured by the OCTANS in the G frame, and $\varepsilon_{wo}^{G}$ is zero-mean Gaussian white noise.

### 2.6. Error Model of DVL

The DVL measures the velocity of the carrier by emitting ultrasonic waves to the seafloor. It is based on the Doppler effect. According to the principle of the DVL, it can provide the velocity of the UUV related to the seafloor. The accuracy of the DVL is affected by many error sources, such as installation errors, scale factor errors, frequency measurement errors, etc. [23]. To simplify the analysis, the errors of the DVL are considered to be composed of the scale factor errors, the random velocity errors, and the white noise. The output of the DVL in the body frame of the DVL (*m* frame), which can be expressed as:

(79) $${\hat{V}}_{d}^{m}=\left(1+\delta K_{d}\right)V_{d}^{m}+\delta V_{d}^{m}+v_{d}^{m},$$

where ${\hat{V}}_{d}^{m}$ is the actual output velocity; $V_{d}^{m}$ is the ideal velocity; $\delta K_{d}$ is the scale factor error of the DVL; $\delta V_{d}^{m}$ is the random velocity error; and $v_{d}^{m}$ is zero-mean Gaussian white noise.

The scale factor error $\delta K_{d}$ is assumed as the random constant and a one-order Markov process can describe the random velocity error $\delta V_{d}^{m}$:

(80) $$\left\{ \begin{array}{l} \delta {\dot{K}}_{d}=0\\ \delta {\dot{V}}_{d}^{m}=-\delta V_{d}^{m}/\tau_{v}+w_{v} \end{array}\right.,$$

where $\tau_{v}$ is the correlation time of Markov process of $\delta V_{d}^{m}$ and $w_{v}$ is the white noise.

The output of the DVL projected in the G frame can be expressed as:

(81) $${\hat{V}}_{d}^{G}=C_{b}^{G}C_{m}^{b}{\hat{V}}_{d}^{m},$$

where $C_{m}^{b}$ is the direction cosine matrix from the m frame to the b frame. The installation error angles are small enough to be neglected. Therefore, $C_{m}^{b}$ is approximate to $C_{m}^{b}=I$.

As $\delta K_{d}$ and $\delta V_{d}^{m}$ can be updated by Equation (80), the velocity of the UUV measured by the DVL in the G frame can be obtained as:

(82) $${\hat{V}}_{d}^{G}=V_{d}^{G}+v_{d}^{G}=C_{b}^{G}C_{m}^{b}V_{d}^{m}=C_{b}^{G}\left[\left({\hat{V}}_{d}^{m}-\delta V_{d}^{m}\right)/\left(1+\delta K_{d}\right)\right],$$

where ${\hat{V}}_{d}^{G}$ is the velocity measured by the DVL after partial error compensation in the G frame; $V_{d}^{G}$ is the ideal velocity of the UUV in the G frame and $v_{d}^{G}$ is zero-mean Gaussian white noise.

## 3. Filter Models of the UUV in Polar Grid Navigation

### 3.1. Dynamic Model

Based on the analysis of Section 2, the attitude errors $\phi^{G}$, the velocity errors $\delta V^{G}$, the position errors $\delta R^{e}$, the gyro drifts $\varepsilon_{c}^{b}$, and the accelerometer bias $\nabla_{c}^{b}$ are chosen as the states to be estimated of the SINS. The gyro constant drift of the OCTANS $\varepsilon_{co}^{o}$ is chosen as the state to be estimated of the OCTANS. The scale factor error of the DVL $\delta K_{d}$ and the random velocity error of the DVL $\delta V_{d}^{m}$ are chosen as the states to be estimated of the DVL. The states of dynamic model based on G frame can be defined as:

$$X={\left[X_{SINS}^{T}\;X_{OCTANS}^{T}\;X_{DVL}^{T}\right]}^{T},\;X_{SINS}={\left[{\left(\phi^{G}\right)}^{T}\;{\left(\delta V^{G}\right)}^{T}\;{\left(\delta R^{e}\right)}^{T}\;{\left(\varepsilon_{c}^{b}\right)}^{T}\;{\left(\nabla_{c}^{b}\right)}^{T}\right]}^{T}\;X_{OCTANS}={\left[{\left(\varepsilon_{co}^{o}\right)}^{T}\right]}^{T},\;X_{DVL}={\left[{\left(\delta K_{d}\right)}^{T}\;{\left(\delta V_{d}^{m}\right)}^{T}\right]}^{T}$$

Based on the attitude error equation (Equation (51)), the velocity error equation (Equation (61)), the position error equation (Equation (73)), the error model of OCTANS (Equation (75)), the error model of DVL (Equation (80)), and assuming that $\varepsilon_{r}^{b}$ and $\nabla_{r}^{b}$ are both zero-mean Gaussian white noise, the differential equations of the states can be described as:

(83) $$\{\begin{array}{lll} {\dot{\phi }}^{G} & = & C_{\omega }{}^{-1}[-(\omega_{iG}^{G}\times )\phi^{G}+(C_{\omega ieR}+C_{\omega eGR})\delta R^{e}+C_{\omega eGv}\delta V^{G}-C_{b}^{G^{\prime }}\varepsilon^{b}]\\ \delta {\dot{V}}^{G} & = & f^{G}\times \phi^{G}+\left[V^{G}\times C_{\omega eGv}-\left(2\omega_{ie}^{G}+\omega_{eG}^{G}\right)\times \right]\cdot \delta V^{G}+\left[V^{G}\times \left(2C_{\omega ieR}+C_{\omega eGR}\right)\right]\delta R^{e}+C_{b}^{G}\nabla^{b}\\ \delta {\dot{R}}^{e} & = & C_{G}^{e}\delta V^{G}-C_{G}^{e}\left(V^{G}\times \right)C_{\varphi R}\delta R^{e}\\ {\dot{\varepsilon }}^{b} & = & 0\\ {\dot{\nabla }}^{b} & = & 0\\ {\dot{\varepsilon }}_{co}^{o} & = & 0\\ \delta {\dot{K}}_{d} & = & 0\\ \delta {\dot{V}}_{d}^{m} & = & -\delta V_{d}^{m}/\tau_{v}+w_{v} \end{array},$$

For simplification of illustration, the dynamic model of the UUV shown in Equation (83) is defined as Model 1. The typical dynamic model of the SINS shown in [24] is defined as Model 2. The dynamic model of the SINS shown in [13] is defined as Model 3. Model 1 and Model 3 are designed for navigation in the polar region, and Model 2 is designed for navigation in non-polar regions. Models 1, 2, and 3 are all based on the principle of traditional SINS. The main difference among Models 1, 2, and 3 is that the *G* frame is chosen as the navigation frame in Model 1, the *g* frame is chosen as the navigation frame in Model 2, and the transversal *g* frame is chosen as the navigation frame in Model 3. Therefore, the meridians’ convergence in high-latitude areas has no impact on Model 1 and 3, while it has an impact on Model 2. The dynamic model of Model 2 can be described as:

(84) $$\{\begin{array}{l} {\dot{\phi }}^{g}=\phi^{g}\times \omega_{ig}^{g}+\delta \omega_{ig}^{g}-C_{b}^{g}\varepsilon^{b}\\ \delta {\dot{V}}^{g}=-\phi^{g}\times f^{b}+\delta V^{g}\times \left(2\omega_{ie}^{g}+\omega_{eg}^{g}\right)+V^{g}\times \left(2\delta \omega_{ie}^{g}+\delta \omega_{eg}^{g}\right)+C_{b}^{g}\nabla^{b}\\ \delta \dot{L}=\frac{\delta V_{N}^{g}}{R_{M}+h}-\delta h\frac{V_{N}^{g}}{{\left(R_{M}+h\right)}^{2}}\\ \delta \dot{\lambda }=\frac{\delta V_{E}^{g}}{R_{N}+h}\sec L+\delta L\frac{\delta V_{E}^{g}}{R_{N}+h}\tan L\sec L-\delta h\frac{V_{E}^{g}\sec L}{{\left(R_{N}+h\right)}^{2}}\\ \delta \dot{h}=\delta V_{U}^{g}\\ {\dot{\varepsilon }}^{b}=0\\ {\dot{\nabla }}^{b}=0\\ {\dot{\varepsilon }}_{co}^{o}=0\\ \delta {\dot{K}}_{d}=0\\ \delta {\dot{V}}_{d}^{m}=-\delta V_{d}^{m}/\tau_{v}+w_{v} \end{array},$$

According to Equation (83), the dynamic models of the UUV based on the *G* frame can be expressed in vector form as:

(85) $$\dot{X}=AX+BW,$$

where A is the system matrix; B is the control matrix; W is the system noise that can be regarded as independent Gaussian white noise $W_{k}∼N\left(0,Q_{k}\right)$; and Q is measurement noise covariance matrix.

$$A=[\begin{array}{lll} A_{15\times 15}^{SINS} & 0_{15\times 3} & 0_{15\times 6}\\ 0_{3\times 15} & A_{3\times 3}^{OCTANS} & 0_{3\times 6}\;\\ 0_{6\times 15} & 0_{6\times 3} & A_{6\times 6}^{DVL} \end{array}],\;A_{15\times 15}^{SINS}=\left[\begin{array}{lllll} A_{1} & A_{2} & A_{3} & A_{4} & 0_{3\times 3}\\ A_{5} & A_{6} & A_{7} & 0_{3\times 3} & A_{8}\\ 0_{3\times 3} & A_{9} & A_{10} & 0_{3\times 3} & 0_{3\times 3}\\ 0_{3\times 3} & 0_{3\times 3} & 0_{3\times 3} & 0_{3\times 3} & 0_{3\times 3}\\ 0_{3\times 3} & 0_{3\times 3} & 0_{3\times 3} & 0_{3\times 3} & 0_{3\times 3} \end{array}\right],\;A_{3\times 3}^{OCTANS}=\left[0_{3\times 3}\right],\;A_{6\times 6}^{DVL}=\left[\begin{array}{ll} 0_{3\times 3} & 0_{3\times 3}\\ 0_{3\times 3} & A_{11} \end{array}\right]\;B={\left[\begin{array}{lll} B_{1} & 0_{3\times 3} & 0_{3\times 3}\\ 0_{3\times 3} & B_{2} & 0_{3\times 3}\\ 0_{15\times 3} & 0_{15\times 3} & 0_{15\times 3}\\ 0_{3\times 3} & 0_{3\times 3} & I_{3\times 3} \end{array}\right]}^{T},\;W={\left[{\left(\varepsilon_{r}^{b}\right)}^{T}\;{\left(\nabla_{r}^{G}\right)}^{T}\;{\left(w_{v}\right)}^{T}\right]}^{T}$$

where, $A_{1}=C_{\omega }{}^{-1}\;(-(\omega_{iG}^{G}\times ))$, $A_{2}=C_{\omega }{}^{-1}C_{\omega eGv}$, $A_{3}=C_{\omega }{}^{-1}(C_{\omega ieR}+C_{\omega eGR})$, $A_{4}=-C_{\omega }{}^{-1}C_{b}^{G^{\prime }}$, $A_{5}=\left(f^{G}\times \right)$, $A_{6}=\left[V^{G}\times C_{\omega eGv}-\left(2\omega_{ie}^{G}+\omega_{eG}^{G}\right)\times \right]$, $A_{7}=V^{G}\times \left(2C_{\omega ieR}+C_{\omega eGR}\right)$, $A_{8}=C_{b}^{G}$, $A_{9}=C_{G}^{e}$, $A_{10}=-C_{G}^{e}\left(V^{G}\times \right)C_{\beta R}$, $A_{11}=-1/\tau_{v}\cdot I_{3\times 3}$, $B_{1}=C_{\omega }{}^{-1}C_{b}^{G^{\prime }}$, $B_{2}=C_{b}^{G}$.

### 3.2. Observation Model

Due to the restriction of the complex underwater environment in the polar region and combining the characters of the UUV, OCTANS and DVL are chosen as the assistants to improve navigation accuracy of the UUV in the polar region. Thus, attitude and velocity errors are chosen as the states to be observed, which can be obtained from the OCTANS and DVL, respectively. Therefore, the observations of UUV are defined as:

$$Z={\left[\begin{array}{cc} {\left(\phi^{G}\right)}^{T} & {\left(\delta V^{G}\right)}^{T} \end{array}\right]}^{T}$$

Based on the analysis in Section 2, the attitude error and the velocity error can be expressed as:

(86) $$Z=\left[\begin{array}{c} {\hat{\Lambda }}_{o}^{G}-\Lambda^{G}\\ {\hat{V}}_{d}^{G}-V^{G} \end{array}\right]=\left[\begin{array}{c} \phi^{G}+\varepsilon_{wo}^{G}\\ \delta V^{G}+v_{d}^{G} \end{array}\right],$$

where $\Lambda^{G}$ and $V^{G}$ are the attitude and velocity of UUV calculated by the SINS, respectively.

The observation model of UUV based on the G frame can be expressed in vector form as:

(87) $$\dot{Z}=HX+V,$$

where H is the observation matrix; $V={\left[\begin{array}{cc} {\left(\varepsilon_{wo}^{G}\right)}^{T} & {\left(v_{d}^{G}\right)}^{T} \end{array}\right]}^{T}$ is the measurement noise vector that is independent Gaussian white noise $V_{k}∼N\left(0,R_{k}\right)$; and R is the measurement noise covariance:

$$H={\left[\begin{array}{llllllll} I_{3\times 3} & 0_{3\times 3} & 0_{3\times 3} & 0_{3\times 3} & 0_{3\times 3} & 0_{3\times 3} & 0_{3\times 3} & 0_{3\times 3}\\ 0_{3\times 3} & I_{3\times 3} & 0_{3\times 3} & 0_{3\times 3} & 0_{3\times 3} & 0_{3\times 3} & 0_{3\times 3} & 0_{3\times 3} \end{array}\right]}^{T}$$

## 4. Filter Algorithm of the UUV in Polar Grid Navigation

The filter algorithm will affect the navigation accuracy. In this section, a modified fuzzy adaptive Kalman filter is proposed to realize the data fusion and to improve the accuracy of the system.

### 4.1. Modified Adaptive Kalman Filter

Based on the Equations (85) and (87), the discrete expression of the filter models are as follows:

(88) $$\left\{ \begin{array}{l} X_{k}=\Phi_{k,k-1}X_{k-1}+\Gamma_{k,k-1}W_{k-1}\\ Z_{k}=H_{k}X_{k}+V_{k} \end{array}\right.,$$

where, $\Phi_{k,k-1}$, $\Gamma_{k,k-1}$ and $H_{k}$ are the discrete expression of A, B and H, respectively.

The traditional adaptive Kalman filter (AKF) for a discrete system of the UUV can be described as [25]:

(89) $$X_{k,k-1}=\Phi_{k,k-1}{\hat{X}}_{k-1}+{\hat{q}}_{k},$$

(90) $$P_{k,k-1}=\Phi_{k,k-1}P_{k-1}\Phi_{k,k-1}^{T}+\Gamma_{k,k-1}{\hat{Q}}_{k-1}\Gamma_{k,k-1}^{T},$$

(91) $$v_{k}=Z_{k}-H_{k}X_{k/k-1}-{\hat{r}}_{k},$$

(92) $$K_{k}=P_{k,k-1}H_{k}^{T}{\left[H_{k}P_{k/k-1}H_{k}^{T}+{\hat{R}}_{k}\right]}^{-1},$$

(93) $${\hat{X}}_{k}={\hat{X}}_{k/k-1}+K_{k}v_{k},$$

(94) $$P_{k}=(I-K_{k}H_{k})P_{k/k-1},$$

where ${\hat{q}}_{k}$ and ${\hat{Q}}_{k}$ are the mean and the covariance of the system noise W, respectively. ${\hat{r}}_{k}$ and ${\hat{R}}_{k}$ are the mean and the covariance of the measurement noise V, respectively. The recursive estimate formulas can be written as:

(95) $${\hat{q}}_{k+1}=(1-d_{k}){\hat{q}}_{k}+d_{k}(X_{k+1}-X_{k+1,k}),$$

(96) $${\hat{Q}}_{k+1}=(1-d_{k}){\hat{Q}}_{k}+d_{k}[K_{k+1}v_{k+1}{\left(K_{k+1}v_{k+1}\right)}^{T}+P_{k+1}-\Phi_{k+1,k}P_{k}{\hat{X}}_{k}\Phi_{k+1,k}^{T}],$$

(97) $${\hat{r}}_{k+1}=(1-d_{k}){\hat{r}}_{k}+d_{k}(Z_{k+1}-H_{k+1,k}X_{k+1,k}),$$

(98) $${\hat{R}}_{k+1}=(1-d_{k}){\hat{R}}_{k}+d_{k}[v_{k+1}v_{k+1}^{T}-H_{k+1}P_{k+1,k}H_{k+1}^{T}],$$

where $d_{k}=(1-b)/(1-b^{k})$, 0<b<1 is the forgetting factor.

In order to simplify the system and keep positive definiteness of ${\hat{Q}}_{k}$ and ${\hat{R}}_{k}$, Equations (96) and (98) are modified as:

(99) $${\hat{Q}}_{k+1}=(1-d_{k}){\hat{Q}}_{k}+d_{k}[K_{k+1}v_{k+1}{\left(K_{k+1}v_{k+1}\right)}^{T}+P_{k+1}],$$

(100) $${\hat{R}}_{k+1}=(1-d_{k}){\hat{R}}_{k}+d_{k}[v_{k+1}v_{k+1}^{T}],$$

where, Q and R are the covariance of the system noise and the measurement noise, respectively.

### 4.2. Fuzzy Inference System (FIS)

The system noise and the measurement noise are assumed to be zero-mean white noise in the conventional AKF, and their covariances are Q and R, respectively. In general, there is no prior information of R changing in different situations [26]. The covariance changes with varied underwater environment and influence the characters of the filter. The smaller R value is the higher weight that the recent measurement is given, and the faster the filter response to the observed values [26,27].

The covariance can be modified by a fuzzy inference system (FIS). The adaptive Kalman filter will achieve the optimal state though the covariance modified by FIS. Therefore, in order to improve the performance of the filter, ${\hat{R}}_{k}$ should be adjusted by FIS:

(101) $$\hat{R}=T{\hat{R}}_{k},$$

where T is the output of the FIS and:

(102) $$V_{k}=Z_{k}-H_{k}X_{k/k-1},$$

where, $V_{k}$ is defined as residual error which reflects the dependence degree of the measurement value to the system model. The residual errors are the differences between the measurements and the predictions of the filter, which can be seen as zero-mean white noise, in general [26]. If the residual errors are not zero-mean white noise, the filter will converge to a large bound, or even diverge. Therefore, the residual errors are used to adjust the filter. The theoretical value of the residual error covariance matrix $M_{v}$, which has association with Q and R, can be described as [28]:

(103) $$M_{v}=H_{k}\left(\Phi P_{k-1}\Phi^{T}+Q_{k-1}\right)H_{k}^{T}+R_{k-1},$$

The actual mean value and covariance matrix of the residual error with j statistical numbers in a period of time can be defined as:

(104) $$\bar{v}=\frac{1}{n}\sum_{j=t-n}^{t}v_{j},$$

(105) $${\hat{M}}_{v}=\frac{1}{n}\sum_{j=t-n+1}^{t}v_{j}v_{j}^{T},$$

where v is the first element in $V_{k}$ to simplify the analysis.

If ${\hat{M}}_{v}$ is much larger than $M_{v}$ and $\bar{v}$ is detached from zero, the filter would be unstable. Thus, $\bar{v}$ and ${\hat{M}}_{v}$ are chosen as the inputs of FIS.

**Figure 2:**
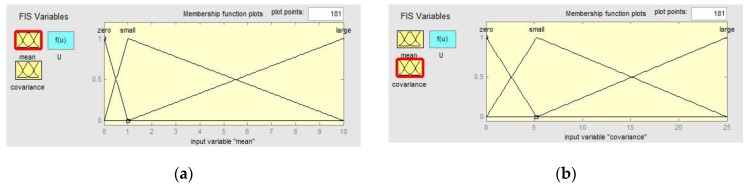
Membership function: (**a**) membership function of input variable “mean”; (**b**) membership function of input variable “covariance”.

The FIS used in this paper is a double-input-single-output FIS, which is based on T-S fuzzy logic. The first elements in the mean $\bar{v}$ and covariance ${\hat{M}}_{v}$ of the residual error are the inputs of the FIS. The output of FIS is T which is used to adjust ${\hat{R}}_{k}$. The triangle membership function is used to blur the inputs. A membership function of inputs can be expressed as Figure 2.

**Figure 3:**
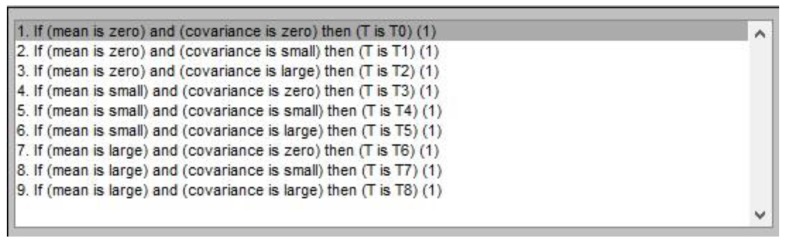
Fuzzy rules.

A total of nine fuzzy rules are used to describe the relationship between the inputs and the output. These fuzzy rules can be shown as Figure 3.

### 4.3. Modified FUZZY ADAPTIVE KALMAN Filter

Based on the analysis above, the whole modified fuzzy adaptive KF is shown as follows:

(106) $$X_{k/k-1}=\Phi_{k,k-1}{\hat{X}}_{k-1},$$

(107) $$P_{k/k-1}=\Phi_{k,k-1}P_{k-1}\Phi_{k,k-1}^{T}+Q_{k-1},$$

(108) $$v_{k}=Z_{k}-H_{k}X_{k/k-1},$$

(109) $$R_{k+1}=(1-d_{k})R_{k}+d_{k}[v_{k+1}v_{k+1}^{T}],$$

(110) $$K_{k}=P_{k,k-1}H_{k}^{T}{\left[H_{k}P_{k/k-1}H_{k}^{T}+T_{k}{\hat{R}}_{k}\right]}^{-1},$$

(111) $${\hat{X}}_{k}={\hat{X}}_{k/k-1}+K_{k}v_{k},$$

(112) $$P_{k}=(I-K_{k}H_{k})P_{k/k-1},$$

(113) $${\hat{Q}}_{k+1}=(1-d_{k}){\hat{Q}}_{k}+d_{k}[K_{k+1}v_{k+1}{\left(K_{k+1}v_{k+1}\right)}^{T}\;+P_{k+1}],$$

**Figure 4:**
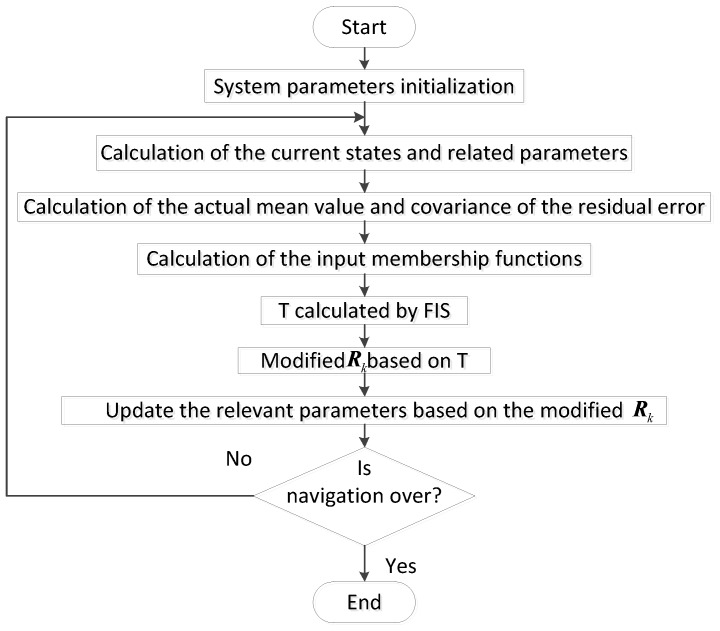
Flow chart of the modified fuzzy adaptive Kalman filter.

In order to describe MFAKF clearly, a flowchart shown in Figure 4 is used to express the process of MFAKF, as shown below.

## 5. Simulations and Experimental Results

Simulations and experiments are conducted not only to verify the effectiveness of the polar grid navigation for the UUV, but also to verify the accuracy improvement of MFAKF compared with AKF. For a simplification of illustration, the SINS/OCTANS/DVL integrated navigation system is labelled as SODINS.

### 5.1. Simulation Results and Analysis

To verify the effectiveness of the proposed algorithm, simulations are realized in the following conditions: Simulation time is 12 h and the filtering period is 0.1 s. The initial position of UUV, including latitude *L* and longitude *λ*, are set as 80° and 126°, respectively.

Sine functions are used to describe the attitudes of the UUV, which include pitch angle, roll angle, and yaw angle. The amplitude of pitch angle, roll angle, and yaw angle are 4°, 5° and 3°, respectively. The period of these angles is 3 s 5 s, and 7 s, respectively. The initial phase of these angles is 0°, 0° and 0°, respectively. The actual misalignment angles are 0°, 0° and 0°, respectively.

The gyro drifts and the accelerometer bias are the two main error sources of SINS [29]. The gyro bias is composed of the gyro constant drifts and the gyro random drifts, which are set as $0.03^{{}^{\circ}}/h$ and ${\left(0.001^{{}^{\circ}}/h\right)}^{2}$, respectively. The accelerometer bias is composed of the accelerometer constant bias and the accelerometer random bias, which are set as $1\times 10^{-6}g_{0}$ and ${\left(1\times 10^{-7}g_{0}\right)}^{2}$, respectively.

The initial conditions of the OCTANS and DVL are set as follows: The gyro constant drifts of OCTANS are set as 0.01°/h and the random drifts are set as ${\left(0.0005^{{}^{\circ}}/h\right)}^{2}$. The velocity random drifts of DVL are set as $\delta V_{dx}^{m}=\delta V_{dy}^{m}=\delta V_{dz}^{m}=0.005\;m/s$, the correlation time of $\delta V_{d}^{m}$ is set as $\tau_{v}=5\;\min$ and the scale factor error is set as $\delta K_{d}=10^{-4}$. To simplify the simulation and for the purpose of this paper, the constant errors of the OCTANS and DVL are assumed to be well compensated, the random errors are assumed well modeled, and white noises are used to describe the measurement noises of the OCTANS and DVL.

The initial state estimation covariance $P_{0}$, system noise covariance Q, and measurement noise covariance R are set shown below.

$$P_{0}=diag\left\{\begin{array}{l} {\left(0.01\pi /180\;\mathrm{rad}\right)}^{2},{\left(0.01\pi /180\;\mathrm{rad}\right)}^{2},{\left(0.01\pi /180\;\mathrm{rad}\right)}^{2},{\left(0.1\;m/s\right)}^{2},{\left(0.1\;m/s\right)}^{2},\\ {\left(0.1\;m/s\right)}^{2},{\left(1\;m\right)}^{2},{\left(1\;m\right)}^{2},{\left(1\;m\right)}^{2},{\left(0.03\pi /180/3600\;\mathrm{rad}/s\right)}^{2},{\left(0.03\pi /180/3600\;\mathrm{rad}/s\right)}^{2},\\ {\left(0.03\pi /180/3600\;\mathrm{rad}/s\right)}^{2},{\left(1\times 10^{-6}g\;{m/s}^{2}\right)}^{2},{\left(1\times 10^{-6}g\;{m/s}^{2}\right)}^{2},{\left(1\times 10^{-6}g\;{m/s}^{2}\right)}^{2},\\ {\left(0.01\pi /180/3600\;\mathrm{rad}/s\right)}^{2},{\left(0.01\pi /180/3600\;\mathrm{rad}/s\right)}^{2},{\left(0.01\pi /180/3600\;\mathrm{rad}/s\right)}^{2},\\ {\left(10^{-4}\right)}^{2},{\left(10^{-4}\right)}^{2},{\left(10^{-4}\right)}^{2},{\left(0.005\;m/s\right)}^{2},{\left(0.005\;m/s\right)}^{2},{\left(0.005\;m/s\right)}^{2} \end{array}\right\}\;Q=diag\left\{\begin{array}{l} {\left(5\times 10^{-4}\pi /180/3600\;\mathrm{rad}/s\right)}^{2},{\left(5\times 10^{-4}\pi /180/3600\;\mathrm{rad}/s\right)}^{2},\\ {\left(5\times 10^{-4}\pi /180/3600\;\mathrm{rad}/s\right)}^{2},{\left(5\times 10^{-6}g{m/s}^{2}\right)}^{2},{\left(5\times 10^{-6}g{m/s}^{2}\right)}^{2},\\ {\left(5\times 10^{-6}g\;{m/s}^{2}\right)}^{2},{\left(0.002\;{m/s}^{2}\right)}^{2},{\left(0.002\;{m/s}^{2}\right)}^{2},{\left(0.002\;{m/s}^{2}\right)}^{2} \end{array}\right\}\;R=diag\left\{\begin{array}{l} {\left(0.01\pi /180\;\mathrm{rad}\right)}^{2},{\left(0.01\pi /180\;\mathrm{rad}\right)}^{2},{\left(0.01\pi /180\;\mathrm{rad}\right)}^{2},\\ {\left(0.01\;m/s\right)}^{2},{\left(0.01\;m/s\right)}^{2},{\left(0.01\;m/s\right)}^{2} \end{array}\right\}$$

To verify the effective of the polar grid navigation, Model 2 and Model 3 are chosen as the comparative models. They all based on traditional SINS. However, different frames are chosen as the navigation frames in these models. Different from Model 2, the traditional g frame is chosen as the navigation frame, the G frame is chosen as the navigation frame in Model 1, and transversal g frame is chosen as the navigation frame in Model 3. The computational time and the memory consumption of the proposed polar grid navigation algorithm are 12327.269780 s and 2164 MB (54%), respectively. The simulation results can be expressed as shown below.

**Figure 5:**
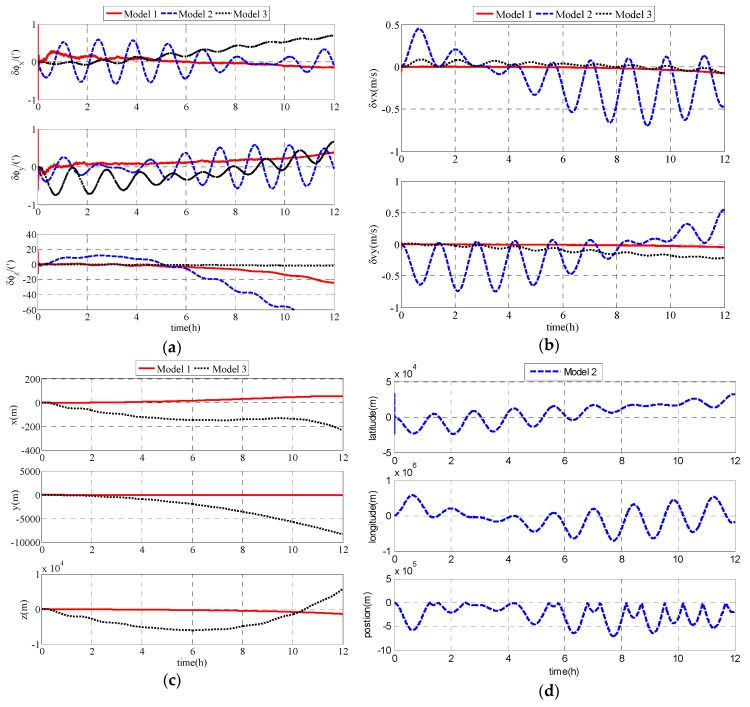
Simulation results: (**a**) attitude errors of the unmanned underwater vehicles (UUV); (**b**) velocity errors of the UUV; (**c**) position errors of Model 1; (**d**) position errors of Model 2.

The coordinate in e frame $R^{e}\left(x,y,z\right)$ describes the position in Model 1. Latitude and longitude describe the position in Model 2. Considering the different expressions of position in Model 1 and Model 2, Figure 5c,d are used to express the position errors, respectively. As shown in Figure 5, compared with Model 2, Model 1 and Model 3 exhibit more accurate performances, and Model 1 is superior to Model 3 in accuracy. Therefore, Model 1 can be effectively used in the polar region.

In order to facilitate comparative analysis, the filter model and the simulation condition are the same for MFAKF and AKF. The estimation errors of SODINS based on MFAKF and KF can be expressed as shown below.

**Figure 6:**
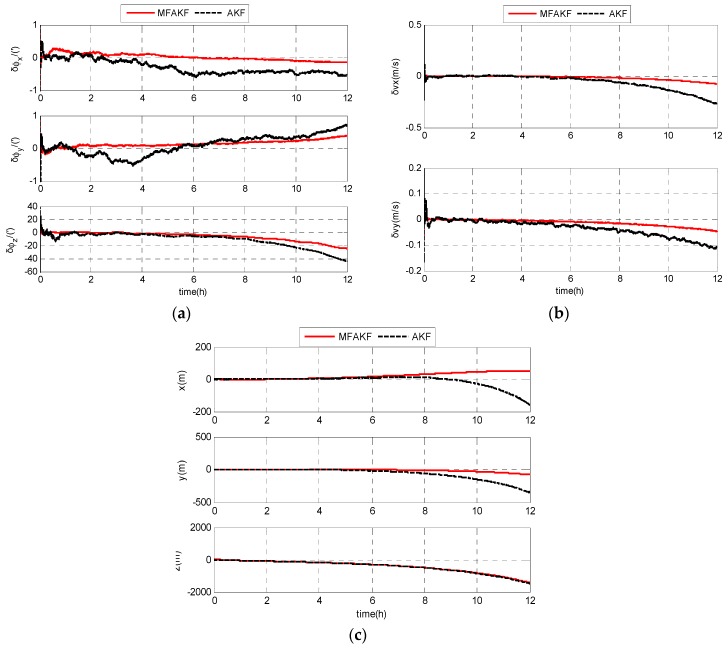
Simulation results of SODINS based on MFAKF and AKF: (**a**) estimation errors of attitude; (**b**) estimation errors of velocity; and (**c**) estimation errors of position.

As shown in Figure 6, the estimation errors of MFAKF is smaller than that of the KF. MFAKF shows better performance than AKF. The RMS estimation errors of SODINS based on MFAKF and KF are shown in Table 1, respectively.

From Table 1, the RMS estimation errors of attitude, velocity, and position based on MFAKF are less than 9.0768’, 0.0256 m/s, and 556.3639 m, respectively. The RMS estimation errors of attitude, velocity, and position based on AKF are less than 14.3786’, 0.0912 m/s, and 573.4550 m, respectively. The results indicate that MFAKF is superior to AKF in the estimation of navigation errors.

### 5.2. Experimental Results and Analysis

With the restriction of the geography, experimental results can be obtained from the semi-physical simulation. The experiment was conducted in non-polar areas, and the measured data is supplied by the IMU. The experimental data in the polar region is composed of the practical measured data and the simulated data. The experimental data includes the angular velocity ${\hat{\omega }}_{ib}^{b}$ and special force ${\hat{f}}^{b}$ in the polar region. The angular velocity in the polar region ${\hat{\omega }}_{ib}^{b}$ is composed of the true angular velocity $\omega_{ib}^{b}$ and the accelerometer bias $\delta \omega_{ib}^{b}$. Similarly, the special force ${\hat{f}}^{b}$ is composed of the true special force $f^{b}$ and the gyro drifts $\delta f^{b}$.

(114) $$\{\begin{array}{c} {\hat{\omega }}_{ib}^{b}=\omega_{ib}^{b}+\delta \omega_{ib}^{b}\\ {\hat{f}}^{b}=f^{b}+\delta f^{b} \end{array},$$

**Figure 7:**
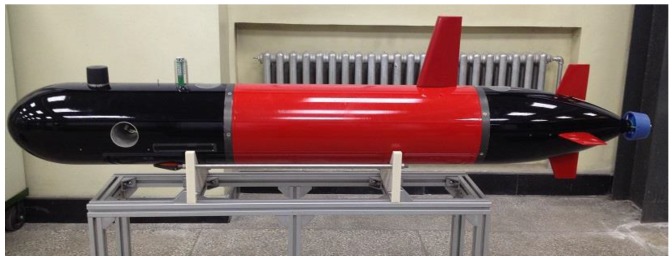
Unmanned underwater vehicle (UUV).

The true values of the angular velocity and the special force can be supplied by the simulation. Meanwhile, gyro drifts and accelerometer bias can be extracted from the practical measured data. Therefore, the practical measured data and the simulated data constitute the experimental IMU data in the polar region. The practical measured data is provided by IMU in the UUV as shown in Figure 7.

This UUV is built by our laboratory. One main rear propeller and three thrusters (including one lateral thruster and two vertical thrusters), one rudder, and one elevator realize the motion control cooperatively. The mission control is realized by the mission control computer. The sensors that we are most concerned with in this paper include the inertial measurement unit (IMU), doppler velocity log (DVL), OCTANS, depth sensor, Global Positioning System (GPS), underwater camera, and sonar. In these sensors, IMU, OCTANS, and DVL play a major role in this paper, while the other sensors are not covered in this paper. The experiment was carried out in a rectangular pool located at the non-polar region (N45°46′ E126°40′). The UUV is ordered to finish the uniform liner motion with a velocity of 1 kn. According to the data collected from the experiment, the gyro drifts and the accelerometer bias can be calculated.

The gyro drifts and the accelerometer bias can be extracted from the practical measured data. The gyro constant drifts are $0.03^{{}^{\circ}}/h$; the gyro random drifts are ${\left(4.094\times 10^{-6}\;\mathrm{rad}/s\right)}^{2}$, ${\left(4.308\times 10^{-6}\;\mathrm{rad}/s\right)}^{2}$ and ${\left(2.386\times 10^{-6}\;\mathrm{rad}/s\right)}^{2}$, respectively; the accelerometer constant bias is $1\times 10^{-6}g_{0}$; and the accelerometer random biases are ${\left(0.00156\;{m/s}^{2}\right)}^{2}$, ${\left(0{.001747\;m/s}^{2}\right)}^{2}$ and ${\left(0.0004063\;{m/s}^{2}\right)}^{2}$, respectively.

The semi-physical simulation was conducted in non-polar areas to overcome the restriction of the geography. Similar with the true values of the angular velocity and the special force of UUV which are supplied by simulation, the signal of the OCTANS and DVL are also provided by the simulation. Based on the characters of the OCTANS and DVL, the relevant parameters of them are set as follows: The OCTANS gyro constant drifts and the gyro random drifts are set as $0.01^{{}^{\circ}}/h$ and ${\left(0.0005^{{}^{\circ}}/h\right)}^{2}$, respectively. The DVL random velocity drifts, the correlation time of $\delta V_{d}^{m}$, and the scale factor error are set as $\delta V_{dx}^{m}=\delta V_{dy}^{m}=\delta V_{dz}^{m}=0.005m/s,$ $\tau_{v}=5\;\min$ and $\delta K_{d}=10^{-4}$, respectively. To simplify the experiment and for the purpose of this paper, the constant errors of the OCTANS and DVL are assumed to be well compensated, the random errors are assumed to be well modeled, and white noise is used to describe the measurement noises of the OCTANS and DVL. Relevant parameters in experiment are the same with those in simulation. In experiment, errors and estimation errors of UUV are also expressed to verify the effectively of the polar grid navigation algorithm.

**Figure 8:**
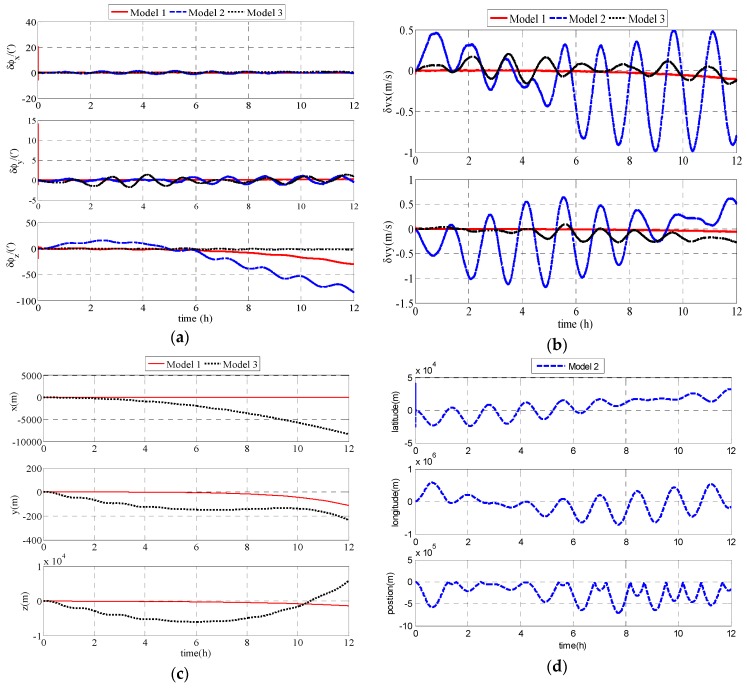
Experimental results: (**a**) attitude errors of the UUV; (**b**) velocity errors of the UUV; (**c**) position errors of Model 1; and (**d**) position errors of Model 2.

As shown in Figure 8, Model 1 is superior to Model 2 and Model 3 in accuracy. The experimental results demonstrate that Model 1 and Model 3 can overcome the problems in Model 2 and Model 1 has better performance than Model 3.

The comparative analysis between MFAKF and AKF in experiment can be expressed as follows.

**Figure 9:**
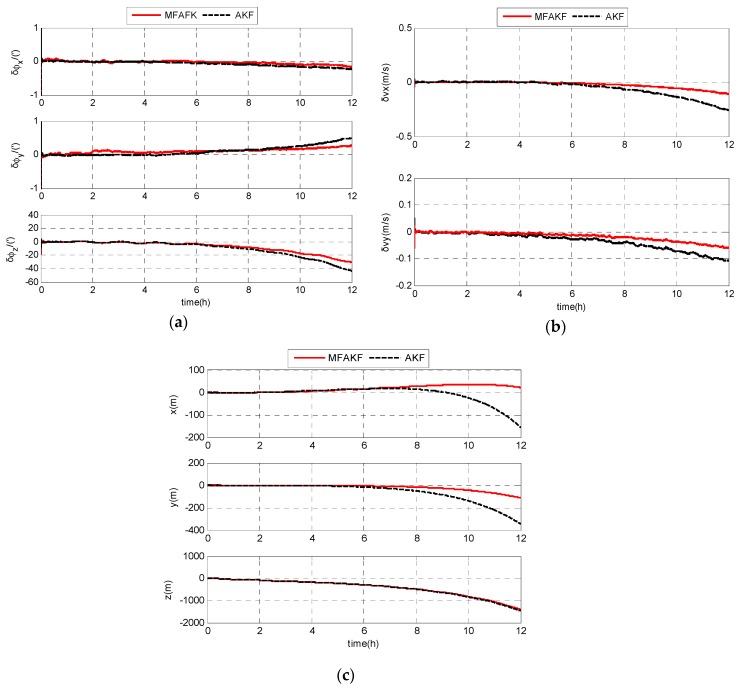
Experimental results of SODINS based on MFAKF and AKF: (**a**) estimation errors of attitude; (**b**) estimation errors of velocity; and (**c**) estimation errors of position.

As shown in Figure 9, MFAKF exhibits better navigation accuracy than AKF. The RMS estimation errors of SODINS based on MFAKF and KF are shown in Table 2, respectively.

From Table 2, RMS estimation errors of the attitude, velocity and position based on MFAKF are less than 11.0893’, 0.0376 m/s and 551.6560 m, respectively. The RMS estimation errors of attitude, velocity, and position based on AKF are less than 15.1294’, 0.0909 m/s and 556.5706 m, respectively. Therefore, the navigation accuracy of SODINS based on MFAKF is better than that based on AKF.

## 6. Discussion

Simulation and experiment results demonstrate that the proposed polar grid navigation can be effectively used on a UUV in the polar region. The comparison between MFAKF and AKF show that the proposed filter algorithm is superior to AKF. To facilitate comparative discussions, the dynamic model of a UUV and the simulation conditions are the same for both MFAKF and AKF. The comparisons among Model 1, Model 2, and Model 3 demonstrate the effectiveness of the polar grid navigation, which can be expressed as shown below. Additionally, compared with the traditional AKF, the advantages of MFAKF are also concluded as follows:

1. SINS is widely used in UUV navigation because of its high autonomy. Model 2 is the typical SINS model for UUVs. It is a general model of SINS and can achieve a navigation accuracy that meets the requirements of UUVs in non-polar regions. While in the polar region, due to the rapid convergence of Earth meridians, there exist calculation overflows and sharply increasing errors in Model 2, in which the geographic frame is chosen as the navigation frame. This is because the error of the upside component of the command angular velocity is related to the tangent value of latitude in traditional SINS. In the polar region, the latitude tends to 90° and the error tends to infinity. Therefore, the geographic frame is not suitable to be chosen as the navigation frame in the polar region. To overcome the problems in the traditional SINS (Model 2) in the polar region, the transversal SINS (Model 3) and polar grid SINS (Model 1) are proposed. The transversal geographic frame and grid frame are chosen as the navigation frame to modify the unsuitability of the traditional SINS, respectively. Although the transversal SINS (Model 3) can realize navigation in the polar region, there are principle errors in the transversal SINS algorithm. The transversal SINS ignores the ellipse of the Earth. Model 1, based on the grid frame, is proposed to overcome the influence caused by the meridians’ convergence in high latitude areas. In the grid frame, the grid planes are parallel with the Greenwich plane. The polar region is the normal region in the polar grid navigation algorithm. There is no impact on the polar grid navigation. Simulation and experiment results also demonstrate that Model 1 is superior to Model 2 and Model 3 in accuracy. The polar grid navigation of UUVs proposed in this paper (Model 1) is suitable for UUV navigating in the polar region.
2. Ignoring the negative terms can not only simplify the filter but can also keep the positive definiteness of the filter. T-S fuzzy logic regulates the residual error close to zero. The covariance is also regulated by FIS to adjust the changing of the environment. The adaptive Kalman filter will achieve the optimal state though the covariance modified by FIS. MFAKF can adjust the changing of the environment. Therefore, MFAKF is superior to AKF in estimating the states of filter.

Based on the analysis above, Model 1 and MFAKF have the better performance than Model 2, 3, and AKF in the polar region. Therefore, the proposed polar grid navigation for UUV can be used in the polar region effectively.

## 7. Conclusions

A polar grid navigation algorithm for UUV is proposed to overcome the unavailability of traditional UUV navigation in polar regions. Considering the complex polar underwater environment and the motion characteristics of UUVs, SINS based on the grid frame with the assistance of OCTANS and DVL is chosen for UUV polar navigation. A modified fuzzy adaptive Kalman filter is used to improve the navigation accuracy. Simulation and experiment results have proven that the proposed polar grid navigation of a UUV can be effectively used in the polar region, and the proposed modified fuzzy adaptive Kalman filter can achieve better accuracy than AKF.

## Figures and Tables

**Table 1:** RMS errors of SODINS in the simulation.

Parameters	MFAKF	AKF
$\phi_{x}$/(′)	0.0872	0.6117
$\phi_{y}$/(′)	0.1429	0.2655
$\phi_{z}$/(′)	9.0768	14.3786
$v_{x}$/(m/s)	0.0256	0.0912
$v_{y}$/(m/s)	0.0182	0.0464
$r_{x}$/(m)	28.2271	37.9480
$r_{y}$/(m)	22.7024	100.8459
$r_{z}$/(m)	556.3639	573.4550

**Table 2:** RMS errors of SODINS in the experiment.

Parameters	MFAKF	AKF
$\phi_{x}$/(′)	0.0571	0.0896
$\phi_{y}$/(′)	0.1257	0.1601
$\phi_{z}$/(′)	11.0893	15.1294
$v_{x}$/(m/s)	0.0376	0.0909
$v_{y}$/(m/s)	0.0241	0.0462
$r_{x}$/(m)	21.3962	36.3067
$r_{y}$/(m)	33.5115	103.3216
$r_{z}$/(m)	551.6560	566.5706
